# New-Onset Monosomy 7-Induced Pancytopenia in a 66-Year-Old Woman

**DOI:** 10.7759/cureus.53159

**Published:** 2024-01-29

**Authors:** Jordan M Brock, Colten Dillinger, David Covey, Jayton A Lim, David E Martin

**Affiliations:** 1 Internal Medicine, Unity Health, Searcy, USA; 2 Graduate Medical Education, Unity Health, Searcy, USA

**Keywords:** acute myeloid leukemia (aml), bone marrow biopsy (bmb), monosomy 7, severe pancytopenia, myelodysplastic syndrome (mds)

## Abstract

Myelodysplastic syndrome (MDS) is characterized by failure to initiate hematopoiesis or impaired maturation of cells, often presenting with pancytopenias with or without associated fatigue, infections, or inappropriate bleeding and bruising. Karyotype analyses of MDS patients commonly show deletion of the q arm of chromosome 7, suggesting loss of this region is likely implicated in the insufficient hematopoiesis seen in MDS. The predisposition to deletion of 7q is commonly inherited, with clinical presentation in early childhood associated with pancytopenia or hematological malignancy. In this case, we present a 66-year-old female who was incidentally found to be pancytopenic in the emergency department while being evaluated for dyspnea, with a bone marrow biopsy later confirming a diagnosis of MDS with monosomy 7. Sporadic loss of 7q can occur at any stage in life without any family history of hematological disease. Our patient has no known personal or family history of MDS, with normal blood counts during hospitalization three years prior, suggesting de novo loss of 7q occurring at greater than 60 years of age.

## Introduction

The prevalence of monosomy 7 seen on karyotype analysis of patients with myelodysplastic syndrome (MDS) or acute myeloid leukemia (AML) suggests that the gene(s) indirectly responsible for hematopoiesis are located in this region [1]. Cytogenetic mapping of these patients reveals variations in the size of the 7q deletion and the specific tumor suppressor genes missing in each case [1]. It is currently thought that this phenotype is caused by an accumulation of multiple gene haploinsufficiencies, potentially including SAMDL9, EZH2, and CUL1 [2,3]. Affected cells are thought to exhibit favored pressure and eventually replace healthy hematopoietic stem cells [2]. Predisposition to these deletions can be inherited in either autosomal dominant, autosomal recessive, or an X-linked pattern [1]. Deletion can also be acquired de novo [2]. Furthermore, the inherited form shows incomplete penetrance, and phenotypic expression is heterogenous [2]. These factors make monosomy 7 widely variable in presentation and age of onset [1]. The most consistent presentation is inherited and presents in pediatric patients with pancytopenia secondary to myelodysplasia or AML [4]. De novo cases are more likely to present in the fourth or fifth decades of life and are also associated with MDS [5]. Additionally, karyotypic abnormalities of chromosome 7q are commonly seen in MDS secondary to chemotherapy [6]. Once monosomy 7 has developed, rapid progression is common, and if transformation from MDS to AML occurs, patients are notoriously treatment-resistant and carry a poor prognosis [1,7]. Effective management requires early diagnosis via bone marrow biopsy, treatment of initial pancytopenia with stimulating factor, and hematopoietic stem cell transplant before leukemic transformation [8].

## Case presentation

We report a case of a 66-year-old female with a history of IV methamphetamine abuse and severe peripheral arterial disease requiring left subclavian stent placement. Four months after her endovascular procedure, she presented to the emergency department for an evaluation of acute generalized chest pain. She also reported six months of severe lower extremity claudication and weakness with worsening dyspnea on exertion. The CTA of the abdominal aorta showed extensive atherosclerotic disease with near occlusion of the distal aorta and iliac arteries with significant collateral and distal reconstitution. The physical exam was negative for hepatosplenomegaly and otherwise benign except for decreased peripheral pulses. Although likely the cause of her claudication and weakness, these atherosclerotic changes were deemed chronic, and no intervention was planned. Acute coronary syndrome was ruled out by serial EKGs and cardiac biomarkers. Laboratory values on admission were significant for severe pancytopenia (Table 1). Chart review showed cell counts were only slightly below normal four years prior to this hospitalization, with normal values eight years prior, suggesting the onset of pancytopenia around 60 years of age. Iron studies were normal other than a mildly elevated ferritin level, and stool guaiac was negative. The hemolytic workup was negative with normal haptoglobin. A peripheral blood smear showed leukopenia with a significant decrease in neutrophils but no definite identifiable blasts, red cells showed some anisocytosis and poikilocytosis with a slight polychromatic shift, and platelets appeared reduced in number but showed no significant morphologic abnormalities. Liver and kidney function tests were normal.

**Table 1:** Serial CBC throughout admission. Two units of packed RBCs transfused between Days 1 and 2. Bone marrow biopsy performed on Day 3. Daily G-CSF was given on Days 4, 5, and 6 CBC: complete blood count, RBCs: red blood cells, G-CSF: granulocyte colony-stimulating factor

	Day 1	Day 2	Day 3	Day 4	Day 5	Day 6	Day 7
White blood cells (4.5-11) thou/µL	3.2	2.9	2.6	2.8	2.8	3.5	5.4
Red blood cells (4.2-5.4) mil/µL	1.7	2.8	2.8	2.7	0.0	2.9	2.4
Hemoglobin (12-16) g/dL	6.0	8.8	8.7	8.6	4.0	8.9	7.5
Hematocrit (37-47) %	17.8	25.9	25.6	25.3	4.0	26.8	23.0
Mean corpuscular volume (81-99) fl	104.0	93.0	91.0	95.0	92.0	93.0	96.0
Mean corpuscular hemoglobin (27-31) pg	34.9	31.7	31.1	32.2	4.3	31.0	31.3
Mean corpuscular hemoglobin concentration (33-37) g/dL	33.7	34.0	34.0	34.0	11.0	33.2	32.6
Red cell distribution width (11.5-14.5) %	20.4	25.3	24.5	23.3	5.8	22.9	22.7
Platelets (150-450) th/µL	51.0	37.0	47.0	34.0	48.0	47.0	45.0
Neutrophils (40-80) %	8.9	6.9	10.6	13.9	4.6	33.2	
Lymphocytes (20-40) %	74.8	74.0	73.8	62.2	24.0	40.9	
Monocytes (0-10) %	11.1	12.3	7.8	16.9	15.0	12.1	
Eosinophils (0-5) %	2.5	2.4	3.9	4.5	0.4	4.3	
Basophils (0-2) %	1.0	1.7	1.6	1.0	1.8	2.0	
Absolute neutrophil count c/µL	250	200	270	280	910	1150	1782

The patient was transfused 2 units of packed RBCs on Day 1 with an appropriate increase in hemoglobin by Day 2. Hematology was consulted and recommended broad-spectrum antibiotic coverage until the absolute neutrophil count (ANC) reached 1000 cells/µL. She was started on filgrastim (granulocyte colony-stimulating factor (G-CSF)) on Day 4 and received three injections (Days 4, 5, and 6), with an improvement in her ANC from 250 to 1,782 cells/µL and subsequent discontinuation of antibiotics by Day 7 (Table 1).

**Figure 1:**
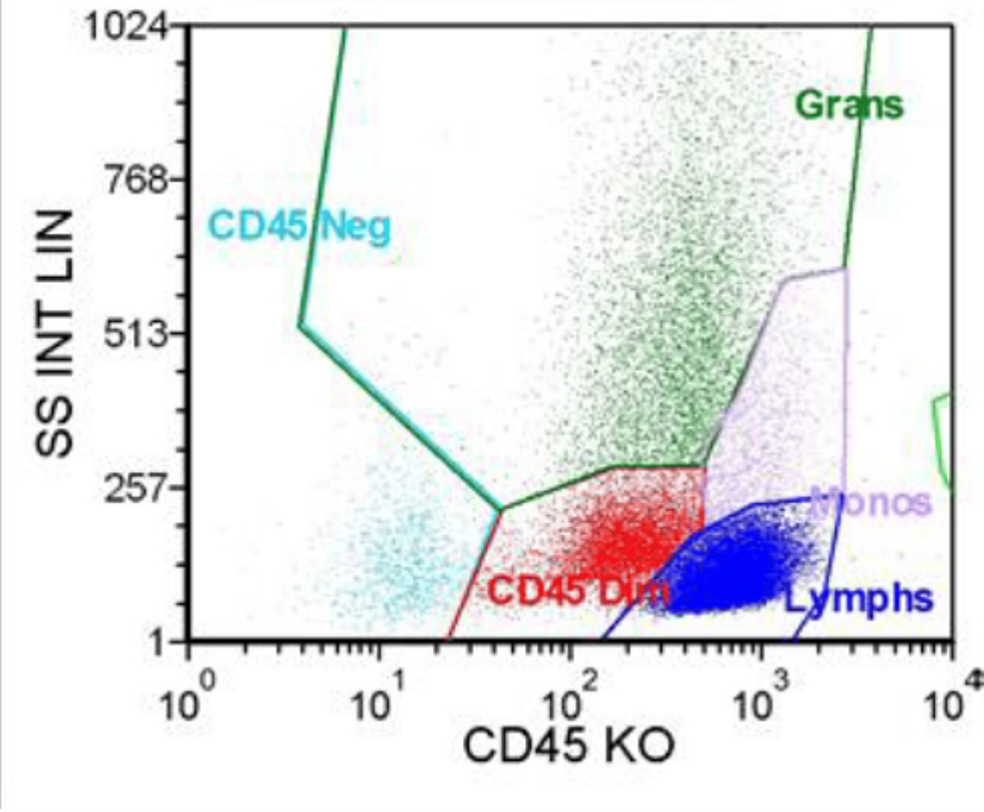
Flow cytometry was performed on the bone marrow aspirate revealing a flow differential of 61.8% lymphocytes, 4.6% monocytes, 15.1% granulocytes, 15.9% CD45 dim (blasts), 2.9% CD45 negative, 0.4% plasma cells, and 8.4% CD34+. Gating on the lymphocyte population reveals predominantly T lymphocytes present with no kappa or lambda light chain restriction and no aberrant markers noted SS INT LIN: side scatter intensity, linear; CD45 KO: CD45 knockout cells

**Figure 2:**
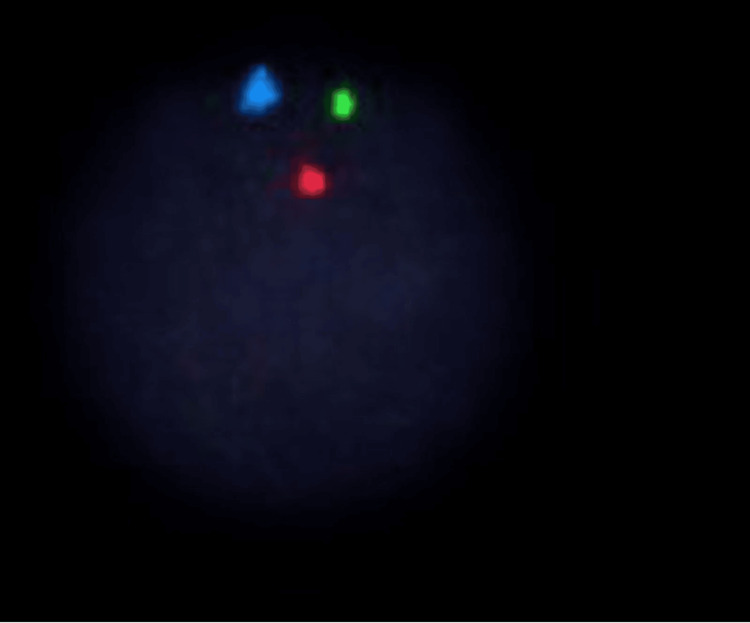
Fluorescence in situ hybridization (FISH) analysis using 7q-/-7 tri: The probe set for chromosome 7 shows an abnormal FISH signal pattern of 1R1G1A indicative of monosomy 7

**Figure 3:**
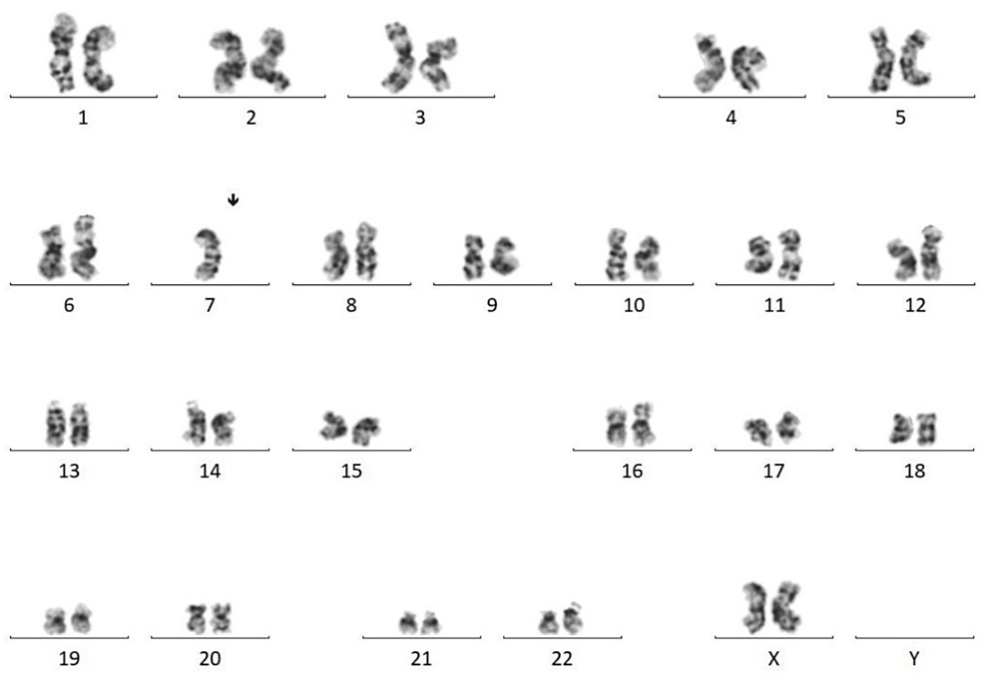
Cytogenetic analysis shows an abnormal female karyotype. All cells analyzed show a loss of one copy of chromosome 7

A bone marrow examination was performed on Day 3 prior to the administration of G-CSF, and the analysis yielded a diagnosis of MDS with myelofibrosis. Flow cytometry was performed on the bone marrow aspirate and was fairly unremarkable (Figure 1). Gating on the lymphocyte population revealed predominantly T lymphocytes present with no kappa or lambda light chain restriction and no aberrant markers noted. MDS fluorescence in situ hybridization (FISH) was performed and revealed that all cells analyzed showed a loss of one copy of chromosome 7 (Figure 2). Cytogenetics final analysis confirmed Karyotype 45, XX,-7(20) (Figure 3).

In the months following her diagnosis, our patient had numerous re-presentations to the hospital for packed RBC transfusions for severe anemia. While awaiting a bone marrow transplant, she ultimately expired five months later from multi-organ failure secondary to her aggressive acute-onset myelofibrosis and likely progression to AML.

## Discussion

MDS is characterized by ineffective hematopoiesis that leads to various cell count abnormalities and maturational dysplasia [9]. Monosomy 7 has been identified as a suspected cause of MDS, with this abnormality seen in 15-25% of cases [9]. Predisposition to the loss of 7q can be inherited and is commonly associated with childhood hematological disease or malignancy; however, it may also occur due to a sporadic, de novo loss of chromosomal material via abnormal chromosomal replication or non-disjunction during cell division [3,9]. Hematopoietic stem cells with monosomy 7 exhibit selective pressure and gradually replace wild-type stem cells, resulting in the development of MDS [1]. The exact genes whose deletion is responsible for the MDS phenotype are unknown, with suspected genes including BRCA2, NF1, DDX4, or XP [10,11]. The pancytopenia associated with MDS can result in life-threatening anemia or infection, and patients with MDS carry significant mortality secondary to progression to AML [1].

Transformation to AML can occur within months, and the prognosis of leukemic transformation from monosomy 7-associated MDS is poor when compared with other AML phenotypes [12]. These cases are notoriously resistant to traditional chemotherapy [8]. Definitive treatment includes hematopoietic stem cell transplantation with or without adjunctive chemotherapy [13]. The relatively rapid progression of leukemic transformation and time-dependent resistance to treatment makes early diagnosis critical to patient prognosis [1]. Of note, biopsy results may not be definitive for monosomy 7 initially, and false negatives can occur secondary to glucocorticoid use [1]. However, FISH and karyotype analysis following a bone marrow biopsy would easily identify monosomy 7 and are the preferred forms of analysis [14].

In cases with a late onset of pancytopenia after age 60, monosomy 7 is most often considered a sporadic mutation rather than an inherited predisposition syndrome [15]. Predisposition syndromes cannot be entirely ruled out without full genetic analysis, but given our patient’s advanced age, suspicion of germline predisposition was low. Predisposition syndromes typically result in the development of MDS or AML during childhood or early adulthood [15]. Furthermore, they are frequently associated with a family history of hematological abnormalities or malignancy [15]. In this patient with no known family history of hematologic disease or malignancy, no predisposing medical condition, or history of chemotherapy treatments, it is likely that her monosomy 7 was acquired sporadically as a de novo mutation.

It is typically appropriate to refer patients with pancytopenia to a hematologist based on their severity and clinical stability. Bone marrow biopsies with flow cytometry and cytogenetic testing are useful and informative, provided they are performed before treatment with recombinant hematopoietic growth factors. Treatment of symptomatic MDS is based on risk and management strategies involving supportive care, hematopoietic stimulating agents, or the initiation of antineoplastic agents like lenalidomide [16].

## Conclusions

The relatively rapid progression of leukemic transformation makes early diagnosis of monosomy 7-driven pancytopenia and MDS a critical step in effective management. Pancytopenia may be caused by a multitude of initiating events, and bone marrow biopsies are sometimes deferred due to the invasive nature of the procedure and patient discomfort. However, delayed diagnosis of myelodysplasia presents a significant increase in mortality. Our patient had poor medical compliance as well as significant social and medical comorbidities, all accounting for potential "red herrings" distracting from her actual pathology. Additionally, the patient's age and lack of history of hematological disease or malignancy served as cognitive hurdles that may have steered the differential diagnosis from chromosomal abnormalities. Diligent workup, including early hematology/oncology consultation and bone marrow biopsy, cannot be understated in the early identification of the etiology of pancytopenia once organic causes have been ruled out. Further research could be done to examine the cost-benefit analysis of bone marrow biopsies in pancytopenic cases.

## References

[1] Olson TS, Dickerson KE, Nakano TA, Wlodarski M (2021). Monosomy 7 predisposition syndromes overview. GeneReviews® [Internet].

[2] Nikoloski G, Langemeijer SM, Kuiper RP (2010). Somatic mutations of the histone methyltransferase gene EZH2 in myelodysplastic syndromes. Nat Genet.

[3] Wong JC, Bryant V, Lamprecht T (2018). Germline SAMD9 and SAMD9L mutations are associated with extensive genetic evolution and diverse hematologic outcomes. JCI Insight.

[4] Wlodarski MW, Sahoo SS, Niemeyer CM (2018). Monosomy 7 in pediatric myelodysplastic syndromes. Hematol Oncol Clin North Am.

[5] Richards S, Aziz N, Bale S (2015). Standards and guidelines for the interpretation of sequence variants: a joint consensus recommendation of the American College of Medical Genetics and Genomics and the Association for Molecular Pathology. Genet Med.

[6] Shannon KM, Turhan AG, Chang SS (1989). Familial bone marrow monosomy 7. Evidence that the predisposing locus is not on the long arm of chromosome 7. J Clin Invest.

[7] Hasle H, Alonzo TA, Auvrignon A (2007). Monosomy 7 and deletion 7q in children and adolescents with acute myeloid leukemia: an international retrospective study. Blood.

[8] Pfeilstöcker M, Tuechler H, Sanz G (2016). Time-dependent changes in mortality and transformation risk in MDS. Blood.

[9] Pezeshki A, Podder S, Kamel R, Corey SJ (2017). Monosomy 7/del (7q) in inherited bone marrow failure syndromes: a systematic review. Pediatr Blood Cancer.

[10] Niemeyer CM, Flotho C (2019). Juvenile myelomonocytic leukemia: who's the driver at the wheel?. Blood.

[11] Aktas D, Koc A, Boduroglu K, Hicsonmez G, Tuncbilek E (2000). Myelodysplastic syndrome associated with monosomy 7 in a child with bloom syndrome. Cancer Genet Cytogenet.

[12] Rentas S, Pillai V, Wertheim GB (2020). Evolution of histomorphologic, cytogenetic, and genetic abnormalities in an untreated patient with MIRAGE syndrome. Cancer Genet.

[13] Locatelli F, Strahm B (2018). How I treat myelodysplastic syndromes of childhood. Blood.

[14] He R, Wiktor AE, Durnick DK (2016). Bone marrow conventional karyotyping and fluorescence in situ hybridization: defining an effective utilization strategy for evaluation of myelodysplastic syndromes. Am J Clin Pathol.

[15] Schratz KE, DeZern AE (2020). Genetic predisposition to myelodysplastic syndrome in clinical practice. Hematol Oncol Clin North Am.

[16] Sekeres MA, Swern AS, Giagounidis A (2018). The impact of lenalidomide exposure on response and outcomes in patients with lower-risk myelodysplastic syndromes and del(5q). Blood Cancer J.

